# Evaluation of feeding practices in the first 12 months of life and nutritional status in an urban setting of a low-resource country: Beira, Mozambique

**DOI:** 10.3389/fnut.2025.1553572

**Published:** 2025-05-15

**Authors:** S. Calgaro, I. Avagnina, A. Vettor, A. Zin, C. Girotto, B. R. Cebola, A. R. Muhelo, I. Rosato, L. Da Dalt, G. Putoto, G. Verlato

**Affiliations:** ^1^Doctor with Africa CUAMM, Beira, Mozambique; ^2^Department of Woman’s and Child’s Health, University of Padova, Padova, Italy; ^3^Azienda Ospedale Università Padova, Padova, Italy; ^4^Department of Pediatrics, Beira Central Hospital, Beira, Mozambique; ^5^Department of Cardio-Thoraco-Vascolar Sciences and Public Health, Unit of Biostatistics, Epidemiology and Public Health, University of Padova, Padova, Italy; ^6^Department of Woman’s and Child’s Health, Pediatric Nutrition Service, Neonatal Intensive Care, University of Padova, Padova, Italy

**Keywords:** malnutrition, children, Mozambique, stunting, nutritional practices

## Abstract

**Background:**

Stunting is a major public health problem, especially in developing countries. In 2020, 37.5% of children under 5 years in Mozambique were stunted.

**Objectives:**

This study aims to describe the nutritional practices in a cohort of Mozambican children and to compare them with international recommendations. The secondary objective is to find differences between chronic malnourished (M) versus non-malnourished (NM) children and to detect factors related to malnutrition.

**Methods:**

A retrospective study was conducted, including children admitted to Beira Central Hospital in Mozambique, using a questionnaire focusing on early nutritional and complementary feeding (CF) practices. We compared the clinical and feeding characteristics of M and NM children and conducted logistic regression to identify factors associated with chronic malnutrition. Data management was performed using Microsoft Excel Office 365 and statistical analysis with Jamovi (version 2.3).

**Results:**

A total of 103 children were studied (median age: 19 months). Seventy percent were exclusively breastfed, 56% continued breastfeeding during CF, but only 8% breastfed until 2 years of age. The introduction of CF occurred at a median age of 6 months, with the main reason being the baby’s crying. Sugar, salt, and sugary drinks were introduced before 1 year of age. At the time of the survey, 42% of the children’s diets were adequately varied. Statistical analysis showed that M children had statistically significant differences in birth weight percentile, were less likely to be breastfed, and consumed fewer dairy products than NM children. Multivariate logistic regression showed that risk factors for chronic malnutrition included HIV infection in both mother and child (OR: 7.5, 95% CI: 1.6–35.09), unaware initiation of CF (OR: 4.35, 95% CI: 1.45–13.05), and birth weight below the 10^th^ percentile (OR: 3.26, 95% CI: 1.02–10.47). In contrast, early and frequent dairy consumption, as well as ongoing maternal breastfeeding during CF, were identified as protective factors.

**Conclusion:**

In our population, the percentage of children with a minimally acceptable diet was low. The use of human milk could be increased, and mistakes in CF practices could be corrected. Our findings highlight the need to raise awareness about the importance of breastfeeding and the timely introduction of appropriately composed CF. Increased attention should be given to children suffering from HIV, with lower birth weights, less breastfeeding, and lower dairy product consumption, in order to prevent malnutrition.

## Background

Globally, malnutrition is considered one of the main public health issues, and its end by 2030 is part of the Sustainable Development Goals (SDGs) of the United Nations (UN) (1). According to the World Health Organization (WHO), malnutrition is defined as a deficiency, excess or imbalance in the intake of energy or nutrients compared to the body’s needs (2). Considering energy or nutrient deficiency, malnutrition can be classified in acute malnutrition (wasting), chronic malnutrition (stunting) and underweight. Wasting refers to a recent and severe weight loss due to an insufficient nutritional intake in a short period or an acute illness that has resulted in rapid weight loss (3). Stunting is the result of chronic or recurrent malnutrition, usually associated with poor socioeconomic conditions, poor maternal health and nutrition, recurrent or chronic illness or inadequate feeding and care of infants and children in early childhood (3, 4). Stunting prevents children from reaching their physical and cognitive genetic potential (5, 6). Chronic malnutrition is caused by inadequate nutritional intake between conception and the first 2 years of life. It is associated both with an altered nutritional status of the mother during pregnancy and breastfeeding and with an altered diet in the first 2 years of life (7).

According to UNICEF, on a global level, in 2022, children under 5 years of age affected by chronic malnutrition were 148.1 million while those with acute malnutrition were 45 million (4); of these, 95 and 97%, respectively, live in Asian and African regions. Despite this, the prevalence of chronic malnutrition on a global level has gradually, albeit slowly, declined over the years, except in Central Africa and Oceania (4).

Mozambique is an African country with a very high prevalence of chronic malnutrition. Even if Mozambique has seen a significant reduction in the prevalence of stunting in children under 5 years, the prevalence remains very high (from 42.6% in 2012 to 36.4% in 2022) (4). In particular, according to the Demographic Health Survey for Mozambique 2022–2023 (8), the prevalence of chronic malnutrition in 2022 was 37%, with regional differences; in particular, the prevalence of chronic malnutrition was higher in the northern regions and lower in the Maputo region. In Sofala province, the prevalence of chronic malnutrition was 30% (8).

In addition, in 2020, the number of children under 5 years who presented acute malnutrition was 3.9% (4). The Mozambican government has made reducing malnutrition a top priority, utilizing both national and international plans to promote food security and nutrition actions in order to support child health and fight malnutrition. For example, some of the focuses of the Multisectoral Action Plan for the Reduction of Chronic Undernutrition in Mozambique 2011–2015 (2020) are the reduction of low birth weight and chronic malnutrition, the promotion of exclusive breastfeeding in the first 6 months of age and to guarantee of at least three meals a day (9). These objectives are also addressed in the National Strategy for Infant and Young Child Feeding (10).

Nonetheless, chronic malnutrition remains at 35%, and it is necessary to conduct regular evaluations and provide additional nutrition education to health professionals in order to enhance the supply chain for nutrition.

Several studies on malnutrition in Mozambique are available, but as far as we know, very few focus on nutritional practices in the first year of life. The study of Marroda et al., conducted in the district of Namuno in Cabo Delgado (Mozambique), evaluated the nutritional practices in the first 2 years of life and found that the WHO indicators were not fulfilled (11).

Furthermore, the DHS data showed that the percentage of children receiving exclusive breastfeeding decreased with the increasing age of the baby, with only one-third of children receiving exclusive breastfeeding in the age group of 4–5 months. Moreover, among children aged 12–23 months, only 68% still had some breastfeeding (8). Regarding complementary feeding (CF), 79% of infants were introduced to solid, semisolid, or soft foods by 6–8 months of age, 15% of breastfed infants aged 6–23 months received a minimal dietary diversity, while 29% received minimal meal frequency and 5% had a minimally acceptable diet (8).

To the best of our knowledge, no studies on this topic have been done specifically in the district of Beira, Sofala Province.

Therefore, the main objective of this study was to analyze the nutritional practices in the first year of life in Beira, Mozambique, particularly focusing on complementary nutrition in a group of children in a low-resource setting, and to compare them to the international guidelines to know which are the critical and the possible improvement points to fight malnutrition.

A secondary objective was to study the nutritional practices in a group of malnourished (M) versus non-malnourished (NM) children.

## Materials and methods

### Study design

This is an observational study that retrospectively described nutritional practices in children during their first year of life.

### Population

Children aged between 6 and 23 months, admitted to the non-intensive care units of the Pediatrics Department of the Central Hospital of Beira (Mozambique), were included in the study. Exclusion criteria were as follows: children suffering from genetic disorders, chronic diseases (apart from HIV infection), or major malformations.

### Setting

The study was conducted at the Pediatrics Department of the Central Hospital of Beira, Mozambique. It is a 1,040-bed referral hospital in the Sofala province, a geographical area that covers approximately 1.7 million people.

### Data collection

Data were collected through a questionnaire administered two times a week between September 2018 and February 2019 to mothers or caregivers of children who met the inclusion criteria. Chronic malnutrition was defined as height-for-age *z*-score <−2, and normal nutritional status was defined as weight-for-length/height *z*-score >−1 and height-for-age *z*-score >−2.

Further data were obtained and/or verified by accessing the patient’s medical record.

The questionnaire was developed based on the 2001 WHO document on CF (12), the 2017 ESPGHAN guidelines on CF (13), and the 2007 WHO document on indicators for assessing infant and young child feeding practices (14).

The questionnaire was not pilot-tested to determine its validity and reliability.

The questionnaire was composed into seven sections (see Supplementary data Sheet).

In the first two sections, demographic data and health information were collected relating to the child (sex, date of birth, gestational age at birth, chronological age, and serological status for HIV infection) and the mother (age, number of pregnancies, number of children, abortions and dead children, and HIV serological status). Children’s HIV status was confirmed by blood polymerase chain reaction (PCR) for HIV.

In the third section, child anthropometric parameters at birth and at the time of the evaluation were recorded. The anthropometric parameters considered were weight, length, and head circumference at birth and at the time of the visit. The weight, expressed in kilograms (kg), was measured using scales Laica^®^; the length, expressed in centimeters (cm), was measured using an infantometer Gima^®^; the head circumference, expressed in centimeters (cm), was measured with a flexible tape measure; the mid-upper arm circumference, measured at the time of the visit, expressed in centimeters (cm), was measured using a special metric bracelet. These measures were then transformed into percentiles using the reference growth charts: Fenton 2003 growth charts (15) for the anthropometric parameters at birth in case of gestational weeks (GWs) < 37; the WHO 2006 growth charts (16) for the anthropometric parameters at birth in case of GWs ≥ 37 and for the anthropometric parameters measured at the time of evaluation. The percentile calculation was performed using “The WHO anthro software for PC, version 3.2.2.”

The fourth part of the questionnaire concerned breastfeeding practices. Data were collected relating to the kind and frequency of milk administered in the first 12 months of life (maternal, formula, or others) and, at the time of investigation, if there was the sterilization of the teats and if the water used for the reconstitution of powdered milk was boiled; finally, the age of discontinuation of breastfeeding was assessed.

The fifth section of the questionnaire concerned CF practices. In the first part, data were collected on how to start weaning (age of introduction of the first food, motivation, and the sequence of introduction of the first foods). Furthermore, the type and frequency of milk consumed daily in conjunction with the CF, and the frequency of non-milk meals during the day, were evaluated. In the second part of the section, information was requested about the time of introduction of numerous types of food (cereals, roots, tubers, legumes and nuts, dairy products, meat, fish, eggs, fruit, vegetables, oil, butter, salt, sugar, spices, water, and sugary drinks).

Through the data collected, adherence to the WHO indicators for the evaluation of feeding practices in early childhood was verified for indicators 2 (exclusive breastfeeding under 6 months), 3 (continued breastfeeding at 1 year), 4 (introduction of solid, semisolid, or soft foods), 5 (minimum dietary diversity), 6 (minimum meal frequency), 7 (minimum acceptable diet), 9 (children ever breastfed), 10 (continued breastfeeding at 2 years), 11 (age-appropriate breastfeeding), 13 (duration of breastfeeding), 14 (bottle feeding), and 15 (milk feeding frequency for non-breastfed children) (14).

### Ethical consideration

The study was approved by the Direction of the Beira Central Hospital (N°729/024.1/GDCP-HCBeira/2017), and we collected data in an anonymous form. We explained the study objectives to the caregivers, and they were free to accept or not to answer the study questionnaire.

### Data analysis

Population characteristics were described using the mean ± standard deviation or the median and interquartile range (IQR; Quartiles I–III).

Statistically significant differences between groups were assessed using the chi-squared test (or Fisher’s exact test for small sample sizes) for categorical variables, and the Mann–Whitney *U*-test for continuous variables. Finally, we performed a multivariable logistic regression with a stepwise method to detect variables associated with chronic malnutrition. We used a stepwise selection approach, combining forward selection and backward elimination. Specifically, we added relevant variables based on a significance threshold of *p* < 0.05. After each addition, we checked whether any included variables should be removed due to *p* > 0.10. The final model was selected when no further changes improved its fit and when it had the lowest Akaike Information Criterion (AIC) value.

Variables considered for inclusion were chosen based on theoretical relevance from the available literature and on variables that were significant in stratified analyses.

Data management was performed using Microsoft Excel Office 365 ^Ⓡ^(Microsoft Corporation) and statistical analysis with Jamovi (The Jamovi project 2023), Jamovi (version 2.3), and Software R (R Core Team 2022).

Significance was set at *p* < 0.05.

## Results

### Descriptive analysis: subjects of the study

A total of 103 children were included in the study (they satisfied the inclusion criteria, and the caregiver accepted to fulfill the questionnaire); the median age at the time of administration of the questionnaire was 19 months (IQR: 15–23.5). Among these, 49 (48%) were male and 54 (52%) were female. The median gestational age at birth was 39 (IQR: 37–40) GWs. The median birth weight was 2,900 g (IQR: 2,600–3,200) with a median percentile of 23 (IQR: 11–58) (Table 1). The mothers of these children had a median age of 25 years (IQR: 21–29), two children (IQR: 1–3), and two pregnancies (IQR: 1–3). Additionally, 17 women (18%) reported having at least one dead child. Overall, 42 (44%) mothers and 24 (24%) children were HIV positive. Anthropometric measures at the time of the study are reported in Table 2.

**Table 1:** Anthropometric data at birth.

	Median value (I–III quartile) at birth	Percentile (I–III quartile) at birth
Weight (g)	2,900 (2,600–3,200)	23.00 (11.00–58.00)
Height (cm)	48.00 (44.00–49.00)	21.45 (5.05–52.40)
Head circumference (cm)	34.00 (32.00–35.00)	35.80 (2.80–66.40)

**Table 2:** Anthropometric data at the time of the study.

	Median value (1^–3^ quartile) at the time of the study	Percentile (1^–3^ quartile) at the time of the study
Weight (g)	8.50 (7.00–10.00)	2.35 (0.00–35.30)
Height (cm)	74.00 (70.00–82.00)	1.30 (0.00–22.27)
Head circumference (cm)	45.50 (44.00–47.00)	24.70 (2.30–64.00)
Arm circumference (cm)	13.00 (12.00–15.00)	6.70 (0.08–53.75)

### Descriptive analysis: nutritional practices

Concerning nutritional practices, 72 children (70%) had exclusive breast milk, 29 (28%) had breast milk and formula milk, and two (2%) had only formula milk until the beginning of CF. Concerning the WHO indicator 2 (12), 55% of subjects practiced exclusive breastfeeding until 6 months of age, 76% continued to have breast milk until 12 months and 8% up to 24 months.

During the CF phase, breast milk was continued by 57 (55%), while 26 (25%) had breast milk and formula, 15 (15%) only formula milk and 5 (5%) did not receive any milk. The median age at which breastfeeding was discontinued was 12.5 months (IQR: 7.20–16).

Only 12 (29%) used boiled water and sterilized the bottle when using formula milk.

The introduction of the first complementary food, made in 43% of cases with millet flour, occurred on average at 6 months (IQR: 5–6). The main reason for starting CF was baby’s excessive crying with breastfeeding alone (61%); the second most frequent reason was the mother’s knowledge of the timing of starting CF (18%). Nonetheless, in some cases, the age of initiation of CF deviated greatly from the WHO recommendations: out of a total of 103 subjects, for two (1.9%) children, the introduction of the first food was at 2 months, for seven (6.8%) at 3 months, and for four (3.9%) at 8 or 9 months.

At the time of the interview, children ate a median of 3 (3–4) meals per day, and 68% used a dedicated plate.

The first food was ‘gruel,’ made using 43% millet flour, 26% with an industrial multicereal product (Cerelac Nestlè), 10% corn flour, 9% rice flour, and 12% other unspecified types of flour.

**Figure 1:**
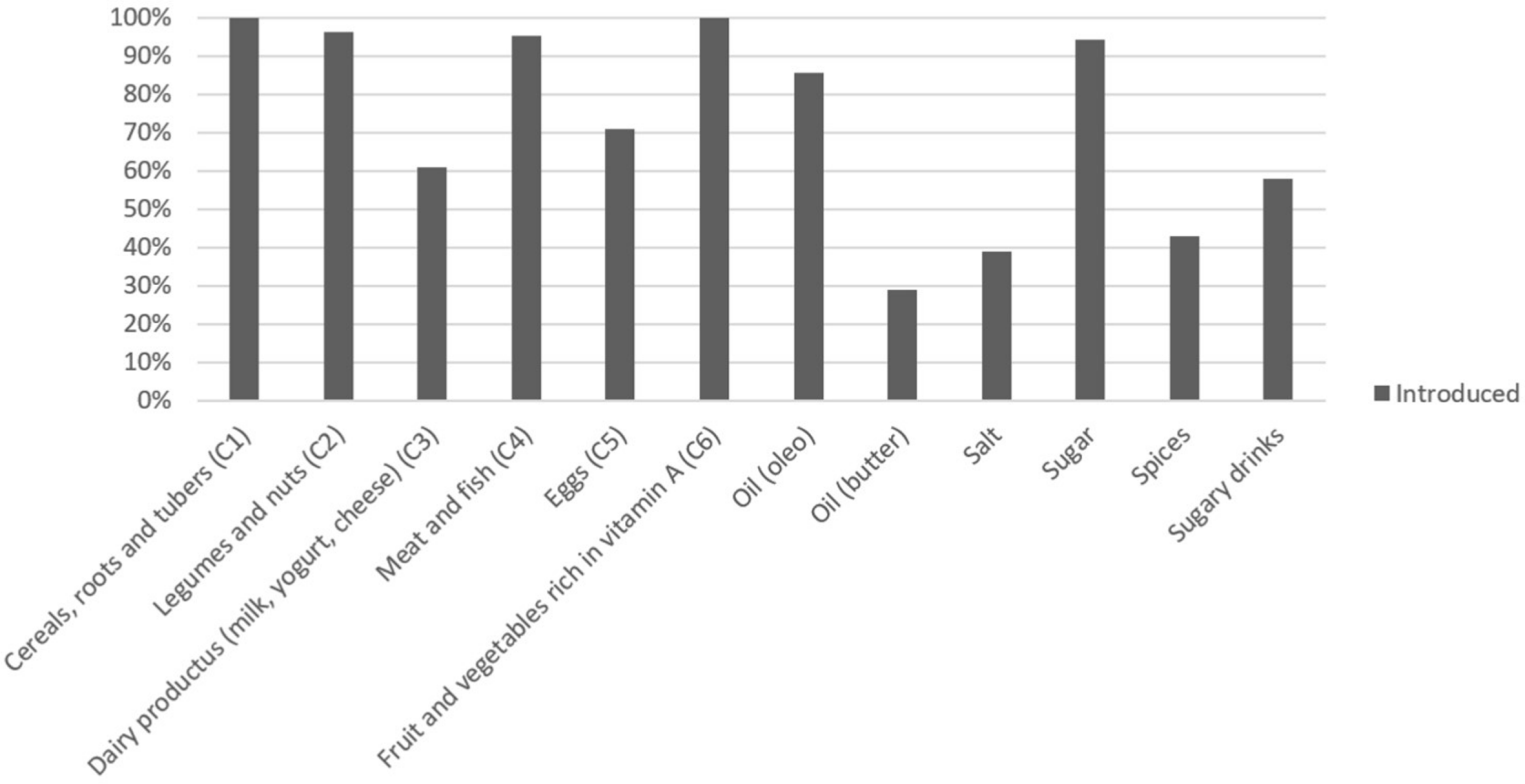
Percentage of children who had already introduced each type of food at the time of the survey.

**Figure 2:**
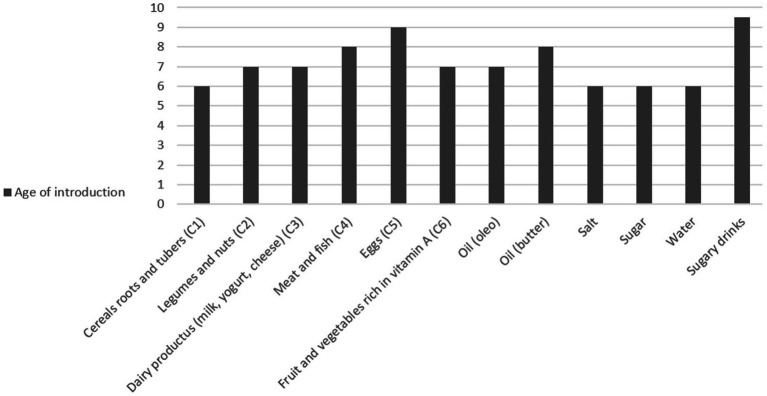
Ages of introduction in months of various type of food.

Figure 1 shows the percentage of children who had already been introduced to each type of food at the time of the survey, and Figure 2 shows the ages of introduction in months of the various types of food.

Cereals, fruits, and vegetables had already been introduced to all children by the time of the interview, but there were greater discrepancies regarding other protein sources. In particular, 4% of children had not yet been introduced to legumes and nuts at the time of the interview, and the same can be said of meat and fish (5%). Eggs were introduced by 70% of children, and dairy products were introduced by 61%. Regarding meat and fish, fish was introduced by a greater number of children than meat, respectively, by 97 (94%) and 80 (77.7%).

Sugar and salt were introduced on average at 6 months, and sugary drinks at 9 months. In particular, sugar was consumed by 94% of children, while sugary drinks by 58%.

The condiment most already introduced was oil (85%), while for butter and olive oil; the percentage of children who had not yet consumed them was higher, 61 and 73%, respectively.

Finally, spices had already been introduced by 43% of children at the time of the interview.

Cereals were introduced at a median age of 6 months, followed by fruit, vegetables, legumes, and dairy products at a median age of 7 months (IQR: 7–9, IQR: 6–9, and IQR: 6–9, respectively). Meat, fish, and eggs were introduced at a median of 9 months (IQR: 7–12 and IQR: 7–12, respectively).

Regarding drinks, water was introduced on average at six (IQR: 5.50–6) months, while soft drinks at 9.50 months (IQR: 8–12) with a median weekly consumption of 2.5 times. Finally, for cow’s milk, data are available for only seven children, but it was introduced on average at 12 (IQR: 9–18) months.

From the analysis of the WHO indicators for the evaluation of feeding practices in infancy, it emerges that 55% of children were exclusively breastfed up to 6 months of age, 76% continued to have breast milk until 12 months, and 8% up to 24 months. The average duration of breast milk intake was 12 months. The minimum frequency of meals was found to be adequate at 80%, dietary diversity at 42%, and a minimum acceptable diet at 36% (Table 3).

**Table 3:** Outcome of the WHO indicators for the evaluation of dietary practices in the 103 subjects of study (2007).

Indicators	% size of the sample*	Results
2-exclusive breastfeeding under 6 months	100%	55%
3-continued breastfeeding at 1 year	93%	76%
4-introduction of solid, semisolid, or soft foods	100%	71%
5-minimum dietary diversity	81.60%	42%
6-minimum meal frequency	98%	80%
7-minimum acceptable diet	83.50%	36%
9-children ever breastfed	84.50%	28%
10-continued breastfeeding at 2 years	36.90%	8%
11-age-appropriate breastfeeding	82.50%	34%
13-duration of breastfeeding	82.50%	12 months
14-bottle feeding	78.60%	30%
15-milk feeding frequency for non-breastfed children	64.10%	33%

*% size of the sample in which the indicator was calculated.

### Comparative analysis: subjects of the study

We then analyzed M versus NM children. NM and M Children were comparable in terms of gestational age at birth and age at the time of the interview (Table 4). Furthermore, no significant differences were found in sex distribution in the two groups (*p*-value 0.35). In both groups, mothers were 25 years old and had two (1–3) children each.

**Table 4:** Gestational age at birth and age at the time of the interview in the two groups.

	M		NM		*p*-value
	Median value (I–III quartile)	Percentile (I–III quartile)	Median value (I–III quartile)	Percentile (I–III quartile)	
Gestational age at birth	39.00	37.00–40.00	39.00	37.00–40.00	1.00
Age at the time of the study (months)	19.00	15.00–23.00	20.00	13.50–25.00	0.47

HIV infection was more present in the group of cases than in the controls: 54% mothers and 35% children versus 30% mothers and 11% children (*p* = 0.01 and *p* = 0.02, respectively).

Birthweight and the relative percentile were statistically significantly lower in cases than in controls: the median weight at birth of children with chronic malnutrition was 2,800 g with a median percentile of 23, while in healthy children, the median values were 3,095 g and 42 (see Table 5).

**Table 5:** Anthropometric data at birth in the two groups.

	M		NM		*p*-value	
	Median value (I–III quartile)	Percentile (I–III quartile)	Median value(I–III quartile)	Percentile (I–III quartile)	Median value	Percentile
Weight (g)	2.80 (2.60–3.00)	23.00 (7.00–34.00)	3.10 (2.80–3.46)	42.00 (23.00–72.00)	0.00	0.01
Height (cm)	47.00 (43.00–49.00)	14.20 (0.82–52.10)	48.50 (48.00–49.00)	31.65 (15.99–77.75)	0.07	0.25
Head circumference (cm)	34.00 (31.20–35.00)	36.00 (5.60–96.30)	34.00 (32.00–34.00)	35.80 (2.60–54.10)	0.91	0.28

### Comparative analysis: nutritional practices

Considering 73% of cases and 66% of controls received exclusive breastfeeding (*p*-value 0.42). Once the introduction of complementary foods began, 55% of cases and 57% of controls continued to have breast milk (*p*-value 0.77). In particular, during weaning, 5% of children with chronic malnutrition did not have any type of milk.

Considering the WHO indicator number 3 (continued breastfeeding at 1 year), 74% of cases and 79% of controls still had breast milk at 12 months (*p*-value 0.53).

The stop of breast milk’s administration occurred at a median (IQ) of 12 (7.50–15.00) months in cases and 13.5 in (7.80–17.20) in controls (*p*-value 0.34). At 2 years, however (the WHO indicator number 10, continued breastfeeding at 2 years), 6% of cases and 10% of controls were still receiving breast milk (*p*-value 0.68).

Crying was the main reason for introducing complementary foods for 71% of cases and 48% of controls (*p*-value 0.61). For both groups, the second most frequent motivation was the mother’s knowledge of the child’s feeding practices, which concerned 13% of cases and 25% of controls (*p*-value 0.18).

**Figure 3:**
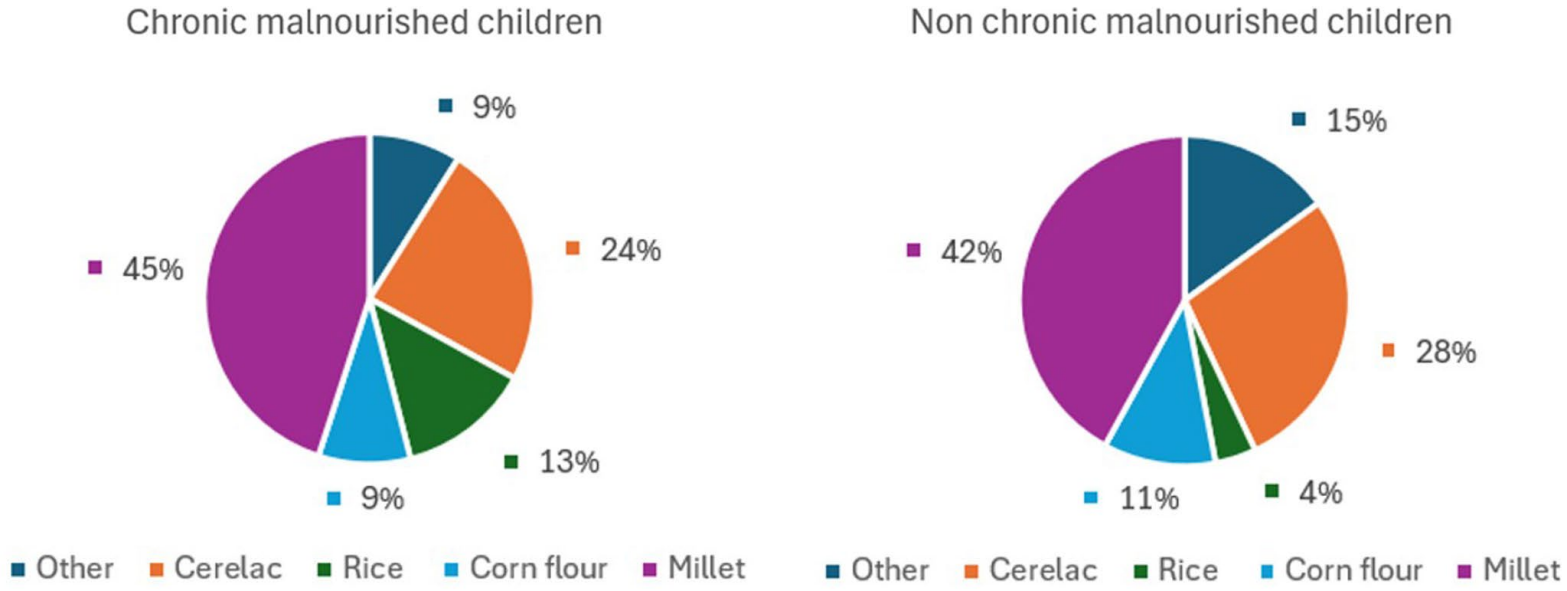
Distribution of the type of flour used in the first food in the two groups (M and NM).

In both groups, the majority of children ate a corn flour-based meal as their first food, and no significant differences were found between the two groups in the distribution of the other flours used (Figure 3).

The study did not highlight statistically significant differences in the percentage and age of introduction of the various classes of foods.

The controls introduced dairy products at a median age of 7 (IQR: 6.0–9.0) months, while cases at a median age of 8.5 (IQR: 6.0–12.0) months. M children ate dairy products 3.0 (IQR: 1.0–7.0) times a week, while NM children 5.0 (IQR: 1.0–7.0) times a week. At the time of the survey, 48% of M and 28% of NM children had not introduced dairy products into their diet yet (*p-value 0.033*).

Water was introduced at a median of 6 months in both M and NM children.

Cow’s milk was introduced to one child in the control group at a median age of 13.5 months, and to three children in the case group at a median age of 12 months.

In both cases and controls, salt, sugar, spices, and soft drinks were, in most cases, introduced before 1 year of age.

At the time of the survey, children ate a median of 3 meals a day in M and 3.5 in NM (*p* = 0.6) and used a dedicated plate in 70 and 65%, respectively (*p*-value 0.58).

Finally, among the WHO indicators for the evaluation of childhood feeding practices, statistically significant differences were found in relation to indicators 9 and 11: the percentages of children who were ever breastfed and had breast milk during the introduction of CF were found higher in controls than in cases (Table 6).

**Table 6:** WHO indicators for the evaluation of childhood feeding practices in cases (M) and controls (NM).

WHO indicators	M		NM		*p-value*
	% values	sample size	% values	sample size	
2-exclusive breastfeeding under 6 months	57%	56	53%	47	0.69
3-continued breastfeeding at 1 year	74%	53	79%	43	0.53
4-introduction of solid, semisolid, or soft foods	70%	56	72%	47	0.76
5-minimum dietary diversity	38%	48	47%	36	0.37
6-minimum meal frequency	78%	55	83%	46	0.58
7-minimum acceptable diet	33%	49	41%	37	0.45
9-children ever breastfed	17%	47	40%	40	**0.02**
10-continued breastfeeding at 2 years	6%	17	10%	21	0.68
11-age-appropriate breastfeeding	20%	46	51%	39	**0.00**
13-duration of breastfeeding	12 months		12 months		0.86
14-bottle feeding	26%	43	34%	38	0.40
15-milk feeding frequency for non-breastfed children	28%	40	42%	26	0.21

### Factors associated with chronic malnutrition

From the multivariable logistic regression, it emerged that risk factors for chronic malnutrition were HIV infection of both mother and child (OR: 7.50, CI: 1.6–35.09, *p* = 0.01) and the initiation of CF without awareness (OR: 4.35, CI: 1.45–13.05, *p* = 0.01). Instead, early and frequent consumption of dairy products (OR: 0.09, CI: 0.02–0.56, *p* = 0.01) and breastfeeding during CF (OR: 0.34, CI 0.10–1.14, *p* = 0.08) were found to be protective factors (Table 7).

**Table 7:** Results from the third multivariate analysis: event = chronic malnutrition (*p* < 0.001).

	Coefficient	Odds ratio (OR)	Confidence interval OR	Significance
Intercept	−0.20	−	−	0.762
HIV				
“Mother only”	0.24	1.27	0.36–4.50	0.714
“Both”	2.02	7.50	1.60–35.09	**0.010**
“Nobody”	−	1	−	−
C3_T3				
“≤8 months and ≥7 times/week”	−2.37	0.09	0.02–0.56	**0.010**
“Other”	−0.56	0.57	0.19–1.74	0.323
“Never”	−	1	−	−
Reason: crying	1.47	4.35	1.45–13.05	**0.009**
Still breastfeeding (yes vs no)	−1.09	0.34	0.10–1.14	**0.081**

## Discussion

Chronic malnutrition early in life is considered a public health problem since it exposes the rapidly growing child to often irreversible adverse outcomes in the short- and long-term periods, such as neurodevelopmental disorders (17, 18), increased susceptibility to infections (19–23) and, in adulthood, metabolic disorders and related diseases (17).

Based on these assumptions, the aim of our study was to analyze the nutritional practices in the first year of life in a group of children in a low-resource setting and to evaluate the adherence to international guidelines. A secondary objective was to study the nutritional practices in a group of M versus NM children.

Relative to breastfeeding practice, among the study population, breastfeeding lasted less than recommended (14).

International societies recommend promoting exclusive breastfeeding for the first 6 months of life (24, 25) or for at least the first 4 months (i.e., 17 weeks, the beginning of the 5th month), while still considering it a desirable goal to continue exclusive breastfeeding until 6 months of age (13). Nonetheless, even after the start of CF, it is recommended that the child continue breastfeeding until 2 years of the child’s life, especially in low-resource countries, as it is a protective factor for health status (13, 24, 25). The WHO global goal for 2025 is to reach breastfeeding for 6 months at least 50% (26), which is in line with our results; nonetheless, it would be extremely important, and it is suggested to continue breastfeeding until 2 years or beyond.

The median age of breastmilk cessation (12.5 months), in fact, deviates from these suggestions (13, 24, 25), and this finding takes on particular importance considering that Mozambique is a low-income country, where poverty also results in poor access to quantitatively and qualitatively adequate food. It is precisely in these cases, in fact, that continuing breastfeeding until at least 2 years of age is important to help meet the child’s caloric and nutritional needs, decreasing the risk of developing malnutrition (13).

Furthermore, we found that only 29% of mothers used boiled water for milk replenishment, and only 29% sterilized their baby’s bottles. Considering the tropical climate and the high prevalence of infectious diseases in Mozambique, the lack of proper hygiene practices contributes to an increased risk of infectious diseases, which, on the one hand, predisposes to a higher risk of developing chronic malnutrition, and on the other hand is inherently more frequent in M individuals, as they are generally immunocompromised.

Concerning CF, we found that while the introduction of CF occurred at a median age of 6 months, there were wide ranges (min 2, max 9 months). Early introduction at 2 or 3 months was motivated by maternal agalactia or erroneous family beliefs. According to a study by Lutter et al. (27), this is associated with several potential risks: incomplete and inadequate infant development to consume food, increased morbidity due to gastrointestinal illnesses if water or food were contaminated, and potential risk of malnutrition since in low-resource countries complementary foods often have a lower nutritional quality than breast milk (24, 27). On the other hand, when the first food was introduced late, parents justified the decision by stating that the child seemed to be satisfied with breastfeeding alone. Late introduction of CF is associated with the inadequate introduction of nutrients essential for growth and development, especially iron, increased risk of food allergies, and decreased likelihood that the child will later accept a wide range of flavors and foods (27, 28).

In our study, mothers’ reasons for initiating CF denoted a lack of education in nutritional requirements. In fact, the main reason for introduction was baby’s crying (61%, or 56 children), as also reported in some studies (29, 30).

From our results, Mozambican children’s first food is ‘gruel’, a soft consistency food composed mainly of flour and water. To make this gruel, most mothers (43%) used millet flour. It is not known whether the choice to use gluten-free flour was made on purpose or was merely due to its availability. In contrast to other traditions, where fruit puree is often the first food (31), the study found that, in Mozambique, the median age of fruit introduction was 7 months.

Considering the composition of the first baby food, it is possible to identify the first two inadequate feeding practices: in the study, in fact, the median age of introduction of sugar and salt was 6 months. In contrast, international societies suggest that neither sugar nor salt should be added to baby’s food (13) and, indeed, their introduction should be delayed to at least 2 years and 1 year, respectively. However, it is important to consider that sugar is a low-priced and readily available food even in low-resource countries. Thus, it could be hypothesized that although there is evidence that, in the long term, high-sugar consumption may have adverse health effects (32), it was introduced for the reasons just mentioned.

At the time of the survey, children were taking a median of 3 (3–4) meals per day and were using a dedicated plate in 68% of cases. Confirming this, the WHO indicator number 6 (14) on minimum meal frequency was positive in 80% of cases. On the other hand, the indicator of minimally acceptable diet [the WHO indicator number 7 (14)] was present in only 36% of the sample, and the indicator of minimal dietary diversity [number 5 (14)] was satisfied in only 42% of cases. In fact, at the time of the survey, the entire group of children with a median age of 19 months had already introduced fruits and vegetables, but the same cannot be said for protein sources. The reasons for all children’s failure to introduce the various protein sources may be a lack of food availability/ affordability or a lack of mother’s knowledge about how the family diet should be composed in order to be nutritionally complete. Our results confirm what has been described among the risk factors for chronic malnutrition: even in cases where caloric needs are met, various other factors can contribute to the onset of this condition, including a diet that is qualitatively poor, not very varied, or with low consumption of animal foods (17).

Considering the age of introduction of various foods, international guidelines (24) do not provide a fixed and precise timing but suggest considering traditions, culture, and ethical or religious choices (13). In addition, usually, it was recommended that cow’s milk should not be used before 12 months of age as it is poor in iron; however, the WHO says that introducing non-human milk after 6 months could be a safe choice if accompanied by complementary foods since the occult blood losses in infants 6–11 months of age are very minor and not likely to affect iron status (33). Gluten should be introduced between 4 and 12 months of age, avoiding large amounts (13). According to the guidelines, allergenic foods can also be introduced after 4 months (17 weeks), without distinguishing between other complementary foods (13, 34–36). Finally, it is interesting to note that 43% of children at the time of the interview had already introduced spices, probably due in part to traditional cooking.

In our study, another important error in feeding concerns soft drinks: although water was properly introduced at a median age of 6 months, that is, in parallel with the start of CF, sugary and carbonated soft drinks were introduced at a median age of 9 months although guidelines advise against their intake at any age but, in particular, during CF. At this age, in fact, the child develops a preference for the tastes to which he or she is most exposed (13).

This second part of the study then involved a comparative analysis between children with chronic malnutrition and children with normal nutritional status. Considering the percentages of both sexes within the groups, it appears that the number of males is higher in cases (52%) than in controls (43%), and the opposite can be said for females. This finding does not reach statistical significance but seems to be in agreement with articles in the literature suggesting a higher prevalence of chronic malnutrition in males (37).

Analysis of anthropometric data at birth shows that the percentiles of cases were statistically lower than controls. In fact, while the median weight of children with chronic malnutrition was 2,800 g with a median percentile of 23, in the healthy, the median values were 3,095 g and a median percentile of 42. The same result was reported for length. The same conclusions could not be drawn for head circumference, as the number of subjects reporting it was too small to allow for the assessment of statistical significance. As expected, considering instead the measurements made at the time of the survey, the differences between the two groups become more pronounced.

The study outlined the higher prevalence of malnutrition in HIV-infected children, an infection that had a prevalence of 7.3% in 2022 in Mozambique (38). In fact, HIV and malnutrition often have a two-way relationship (39–44), where each condition interacts with and exacerbates the other, increasing vulnerability to infection and further worsening health status (43). HIV can be transmitted from mother to child during pregnancy, childbirth and/or breastfeeding (44, 45). In order to protect the health of both mother and child, in 2016, the WHO published guidelines on this issue (24), which emphasized that pharmacologically treated HIV infection is not a contraindication to pregnancy or even breastfeeding and, indeed, that breastfeeding is recommended even up to 2 years of age or at least until supplementary feeding alone can provide sufficient amounts of energy, macro- and micronutrients to the child. In case the mother is HIV-positive, to prevent transmission of the infection to the child, the child is given drugs from birth until 6 weeks of age if the mother is taking antiretroviral therapy or until 1 week after breastfeeding stops if the mother is not taking the therapy (46).

Considering breastfeeding and CF practices, it is interesting to note that the comparative analysis did not show statistically significant differences in most of the variables investigated, with the exception of two WHO indicators (14) for the evaluation of feeding practices. The first indicator relates to the percentage of children under 2 years of age who always received breast milk, and the second indicator relates to the adequacy of breast milk intake in relation to other foods in children under 2 years of age. Both indicators were significantly lower in the case group, which seems to imply that among the protective factors toward chronic malnutrition, breastfeeding carried out in a continuous and prolonged manner may also be considered. In this regard, although studies available in the literature have drawn conflicting conclusions (47–51), the WHO maintains that it is good practice to breastfeed exclusively during the first 6 months, continuing until 12 months and hopefully until 24 months or as long as breastfeeding is agreeable and safe for both baby and mother (13). The rationale for these recommendations lies in the innumerable benefits that seem to be associated with breastfeeding for both mother and baby. For example, benefits for the baby include protection from infections [especially gastrointestinal (24), respiratory, and ear (52, 53)], improved intestinal microflora (54), and the resulting decrease in mortality from all causes, from diarrhea (55), that among M infants (24), and, if breastfeeding within an hour of birth, even neonatal mortality in general (24, 56). The same benefits have also been found with complementary breastfeeding, although obviously to a lesser extent than with exclusive breastfeeding (19). Despite this, exclusive breast milk intake is still suboptimal, so much so that one of the WHO goals for 2025 is to increase the percentage of exclusive breastfeeding to at least 50% (57).

No significant differences were found in any of the WHO indicators (55) related to CF, probably because of the low numerosity of the sample, but, nevertheless, in the group of children with chronic malnutrition, the percentages concerning minimal dietary diversity, minimal food frequency (indicator number 6) and minimally acceptable diet are lower. Children with chronic malnutrition have a less varied and adequate diet and consume fewer meals during the day; these data need to be taken into consideration. Statistically significant differences in CF practices were detected in the frequency of weekly consumption of dairy products, which was higher in controls than cases. It can be inferred that this is due to the good nutritional values of milk and dairy products: these provide protein, minerals, vitamins, and fatty acids and, in the case of yogurt, also probiotics, which are protective against infections (58). However, it should be considered that milk and dairy products are not typical Mozambican products, so they may be available only to those with greater economic resources or greater awareness regarding the beneficial effects.

Compared to the data in the literature, this study confirmed low-birth-weight percentile as a risk factor (59, 60) for chronic malnutrition. Nonetheless, regarding low birth weight, applying multivariate logistic regression, a weight of less than 10 percentile exposes a 3.56 times higher risk of chronic malnutrition than subjects with normal anthropometric parameters (OR: 3.56). However, it is very interesting that although statistical significance is not reached, those born with a weight percentile between 11 and 25, and less considered, are also exposed to an increased risk of chronic malnutrition (2.5 times).

Our study outlines that low birth weight is thus one of the major risk factors for the development of chronic malnutrition (59, 60) and that being under 25° percentile could be a risk factor. Nutritional deficits during fetal life increase the risk of maintaining this issue throughout life. This appears to be related to the nutritional status of the mother during the gestational period: pregnant women with chronic malnutrition generate offspring with lower birth weight (61–63). Children suffering from HIV together with their mother had a risk of 7.5 times being M, as those with a birth weight below 10° percentile (3.26 times) and having a mother not prepared to start CF (4.25 times). In contrast, the mother’s age of less than 20 years, often associated with an increased risk of malnutrition (46), was not found to be a risk factor in this study.

The start of CF due to infant crying increased the risk by more than 4-fold. Therefore, this underscores the need to intensify nutrition educational projects and counseling. The introduction of dairy products <8 months is instead a protective factor. Interestingly, the WHO stated that infants 6–11 months of age fed milk other than breast milk, either milk formula or animal milk, can be fed (conditional, low-certainty evidence). This, in fact, could be due to the consideration that the risk of malnutrition would overcome the risk of anemia, which is also reduced in children >6 months (33).

It would be useful to consider implementing nutritional interventions in pregnancy and once the baby is born in order to improve the nutritional status of pregnant women and also to increase awareness of the importance of breastfeeding and nutrition in the first years of life (in particular, during CF).

Our study has some limitations; in particular, the limited number of patients could not have allowed us to find more statistically significant differences between chronic M and NM children, though this was a secondary objective. Another limit could be the self-reporting of some of the responses.

## Conclusion

In our population, while complementary nutrition started at an adequate age, there was a wide range in the timing of introduction. Furthermore, the percentage of children with a minimally acceptable diet was low, feeding with human milk could be increased, and CF mistakes (early introduction of sugar/salt and soft drinks introduction) should be corrected.

Our findings highlight the need to raise awareness of the importance of timely and appropriate CF composition and that in order to prevent malnutrition, human milk feeding should be expanded. The attention on children suffering from HIV, with lower birth weight, and with less breastfeeding and dairy product consumption should be increased to prevent malnutrition.

Additional interventions focusing on nutritional practices and maternal education will help us establish practices that improve children’s nutritional status.

## Data Availability

The raw data supporting the conclusions of this article will be made available by the authors, without undue reservation.
