# Supplementary material for: Evaluation of feeding practices in the first 12 months of life and nutritional status in an urban setting of a low-resource country: Beira, Mozambique
Source: Front Nutr. 2025 May 15;12:1553572. doi: 10.3389/fnut.2025.1553572 (PMC12119307; doi:10.3389/fnut.2025.1553572)
Supplement: Supplementary file 1 [file Data_Sheet_1.pdf]

QUESTIONÁRIO DE AMAMENTAÇÃO E ALIMENTAÇÃO COMPLEMENTAR

Data............................... Serviço…...........................

A. DADOS GERAIS DA CRIANÇA

Dia de nascimento : …/…/…..
Idade gestacional : …………………..
Idade no dia do internamento :………

Sexo : □ masculino □ feminino
HIV criança: □ pos □ neg

B. DADOS DA MÃE

Idade da mãe:………………………...
Gesta……..
N° filhos : ………………..
N° filhos falecidos……………………

HIV mãe: □ pos □ neg

C. DADOS ANTROPOMÉTRICOS/CLÍNICOS

1. **Peso:**
   Peso ao nascimento ………. g Percentil: ……….
   Peso no dia do internamento ..……. kg Percentil: ……….

2. **Altura:**
   Altura ao nascimento ………. cm Percentil: ……….
   Altura no dia da consulta ………. cm Percentil: ……….

3. **Perímetro craniano:**
   Perímetro craniano ao nascimento ………. cm Percentil: ……….
   Perímetro craniano no dia do internamento.……. cm Percentil: ……….

4. **Perímetro braquial no dia do internamento** ………. cm Percentil: ………

5. **Doenças relevantes** (em curso ou recentes):
   …………………………………………………………………………………………
   …………………………………………………………………………………………

D. AMAMENTAÇÃO

1. A criança tomou leite materno? □ sim □ não
   Se sim, desde …………………………… até…………………………
2. A criança tomou leite artificial? □ sim □ não
   Se sim, desde …………………………… até…………………………
3. Se a criança tomou leite artificial, como você preparou o leite?

a) Ferveu a água? □ sim □ não

b) Ferveu o biberon? □ sim □ não

4. A criança tomou outro tipo de leite? □ sim □ não

   Se sim, desde …………………………… até…………………………
   Se sim, qual tipo de leite?...

5. Até que idade o bebê tomou o leite materno?..

6. Que leite está a tomar a criança agora?

   □ Leite materno

   □ Leite artificial

   □ Leite de vaca

   □ Outros (qual?..........................)

   □ nenhum

7. Quantas vezes por dia a criança toma leite (não leite materno) agora? ………………….

E. ALIMENTAÇÃO COMPLEMENTAR

1. A que idade a criança começou a alimentação complementar?......………………………..
2. Porque você introduziu outros alimentos?

   ....
3. Qual foi o primeiro alimento que introduziu?...
4. O que você usou para preparar a primeira papa durante o desmame?

   ....

   ....
5. Como você introduziu outros alimentos?

   I. ....

   II. ....

   III. ....

   IV. ....

   V. ....

   VI. ....

6. Que leite tomou a criança durante o desmame?

   □ Leite materno

□ Leite artificial (qual? ....)

□ Outro leite (qual? ....)

7. Quantas vezes por dia o bebê tomou o leite durante o desmame (até 1 ano)?

□ <3 vezes □ 3-6 vezes □>6 vezes

8. Quantas vezes a criança come atualmente por dia? ……………………………………….

	**Idade da introdução**
**Cereais, raízes e tubérculos** (milho, arroz, trigo, messe, mapira, tapioca, batatas, mandioca, batatas doce, etc.)	
**Leguminosas e fruta com casca** (feijões, amendoim…)	
**Leite e derivados**	
**Carne** (frango, vaca, porco, etc.)	
**Peixe, lulas, camarão, caranguejo**	
**Ovos**	
**Fruta** (banana, manga, papaia, ananás, maçã, malambe, limão, laranja, tangerina, etc..)	
**Hortícola** (couve, beringela, abóbora, pepino, tomate, cenour, repolho, etc..)	
**Óleo/azeite**	
**Manteiga, margarina**	
**Temperos**	
**Sal**	
**Açúcar**	
**Água**	
**Refrescos**	
**Sumo**	
